# Supplementary figures and images for: Exploring the unmapped DNA and RNA reads in a songbird genome
Source: BMC Genomics. 2019 Jan 8;20:19. doi: 10.1186/s12864-018-5378-2 (PMC6323668; doi:10.1186/s12864-018-5378-2)

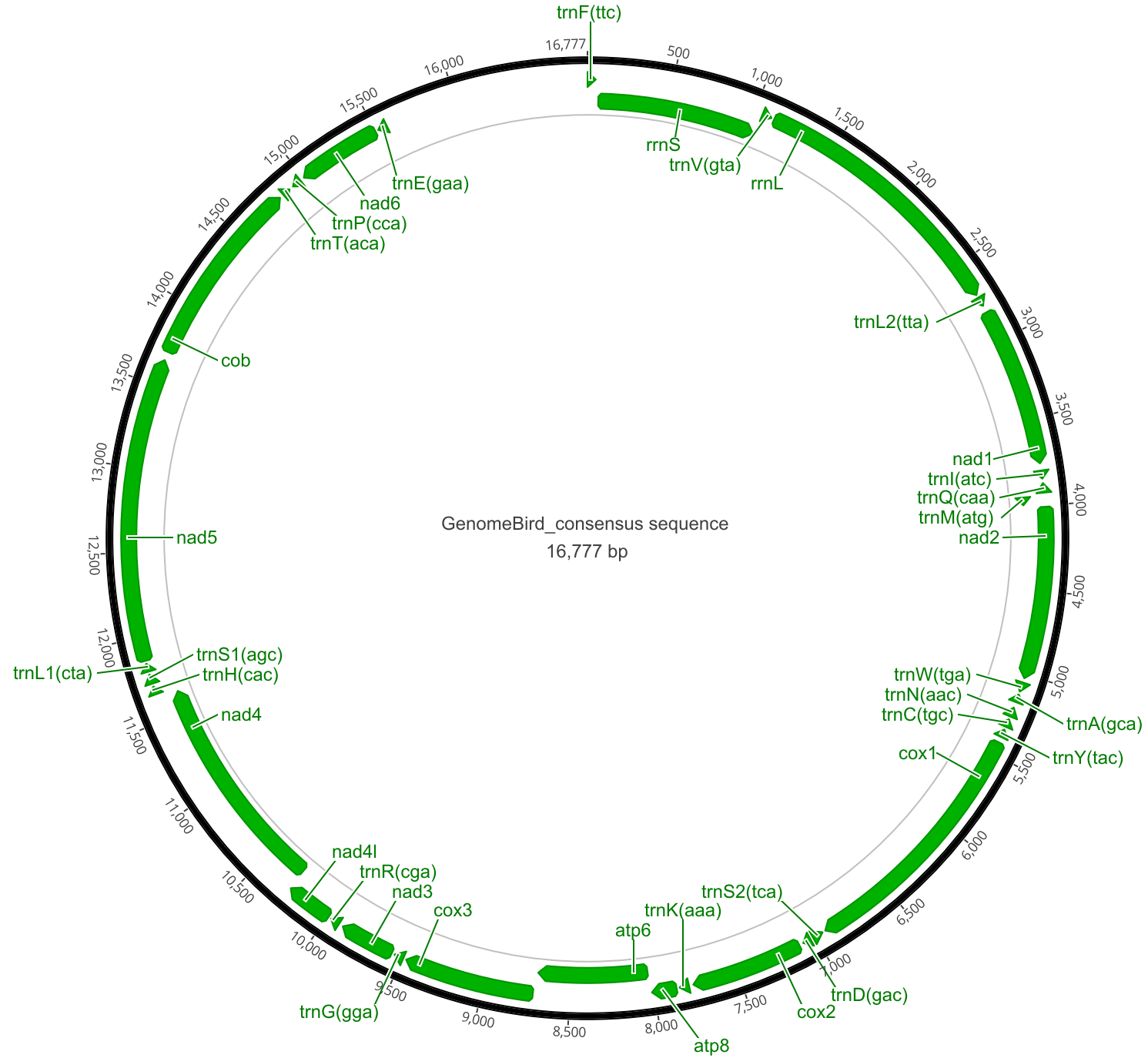

Supplement: Supplementary file 2 — Figure S1. Newly constructed and annotated mitochondria of the reference bird. (PDF 125 kb) [file 12864_2018_5378_MOESM2_ESM.pdf]

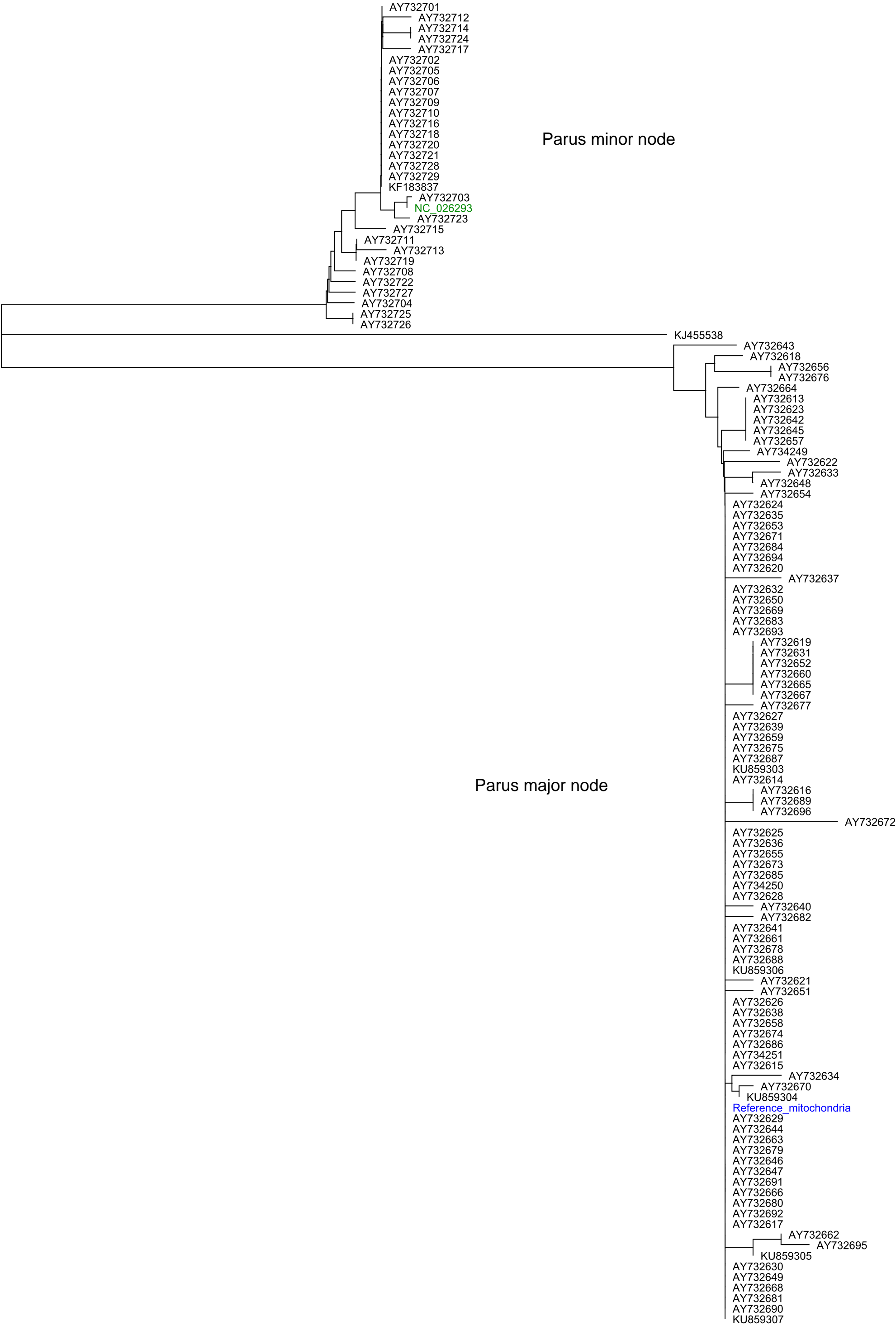

Supplement: Supplementary file 3 — Figure S2. Neighbour-Joining phylogenetic tree between the newly constructed mitochondria (blue), the already existing reference mitochondria (green) and 123 sequences of NADH dehydrogenase subunit 2 (ND2) gene from both Parus major and Parus minor. (PDF 228 kb) [file 12864_2018_5378_MOESM3_ESM.pdf]
